# Clinical and treatment-related predictors of complete response after total neoadjuvant therapy for rectal cancer in a large multicenter analysis

**DOI:** 10.1016/j.ctro.2026.101120

**Published:** 2026-02-05

**Authors:** Georg W. Wurschi, Melanie Schneider, Jan-Niklas Becker, Bernd Frerker, Samuel M. Vorbach, Felix Ehret, Markus Diefenhardt, Fabian Schunn, Maria-Elena von Gruben, Marcel Büttner, Elgin Hoffmann, Alexander Rühle, Josephine Beier, Simone Ferdinandus, Maike Trommer, Ezgi Ceren Sahin, Julian Hlouschek, Kynann Aninditha, Daphne Schepers von Ohlen, Justus Kaufmann, Alina Depardon, Hai Minh Ha, Christopher Kessler, Adrianna Cieslak, Simon Trommer, Alexander Fabian, Mathias Sonnhoff, Florian Rißner, Maximilian Römer, Klaus Pietschmann

**Affiliations:** a Department of Radiotherapy and Radiation Oncology, Jena University Hospital, Jena, Germany; b Comprehensive Cancer Center Central Germany (CCCG), Partner site Jena, Jena, Germany; c Interdisciplinary Center for Clinical Research (IZKF), Jena University Hospital, Jena, Germany; d Department of Radiotherapy and Radiation Oncology, Faculty of Medicine and University Hospital Carl Gustav Carus, Technische Universität Dresden, Dresden, Germany; e Department of Radiotherapy, Hannover Medical School, Hannover, Germany; f Department of Radiotherapy and Radiation Oncology, Rostock University Medical Center, Rostock, Germany; g Department of Radiation Oncology, Medical University of Innsbruck, Innsbruck, Austria; h Charité – Universitätsmedizin Berlin, Corporate Member of Freie Universität Berlin and Humboldt-Universität zu Berlin, Department of Radiation Oncology, Berlin, Germany; i German Cancer Consortium (DKTK), partner site Berlin, a partnership between DKFZ and Charité – Universitätsmedizin Berlin, Germany; j Department of Radiotherapy and Oncology, University Hospital, Goethe University Frankfurt, Frankfurt am Main, Germany; k Frankfurt Cancer Institute (FCI), Frankfurt am Main, Germany; l Department of Radiation Oncology, University Hospital Heidelberg, Heidelberg, Germany; m Department of Radiotherapy and Radiation Oncology, University Hospital Hamburg-Eppendorf, Hamburg, Germany; n Department of Radiation Oncology, University Hospital Tübingen, Tübingen, Germany; o German Cancer Consortium (DKTK) Partner Site Tübingen, Tübingen, Germany; p German Cancer Research Center (DKFZ), Heidelberg, Germany; q Department of Radiation Oncology, Medical Center – University of Freiburg, Faculty of Medicine, University of Freiburg, Germany; r Department of Radiation Oncology, University Medical Center Leipzig, Leipzig, Germany; s Comprehensive Cancer Center Central Germany (CCCG), Partner site Leipzig, Leipzig, Germany; t Department of Radiation Oncology, Cyberknife and Radiotherapy, Faculty of Medicine and University Hospital Cologne, Köln, Germany; u Center for Integrated Oncology Aachen Bonn Cologne Duesseldorf (CIO ABCD), Köln, Germany; v Department of Radiotherapy, West German Cancer Center, University Hospital Essen, Essen, Germany; w Department of Radiooncology, Klinikum Stuttgart, Stuttgart, Germany; x Department of Radiotherapy, University Medical Center Schleswig-Holstein/Lübeck, Lübeck, Germany; y Department of Radiooncology, University Medical Center of the Johannes-Gutenberg-University Mainz, Mainz, Germany; z Department of Radiation Oncology, Universitätsklinikum Erlangen, Friedrich-Alexander-Universität Erlangen-Nürnberg, Erlangen, Germany; a1 Department of Radiation Oncology, Otto von Guericke Universität Magdeburg, Magdeburg, Germany; a2 Department of Radiation Oncology, Technical University of Munich (TUM), School of Medicine and Klinikum Rechts der Isar, München, Germany; a3 Department of Radiation Oncology, University Medicine Mannheim, Medical Faculty Mannheim, Heidelberg University, Mannheim, Germany; a4 Center for Radiotherapy and Radiation Oncology, Bremen, Germany; a5 Department for Radiotherapy, University Hospital Halle, Halle (Saale), Germany; a6 Department of Radiation Oncology, University Hospital Schleswig-Holstein Campus Kiel, Kiel, Germany; a7 Center for Clinical Studies, Jena University Hospital, Jena, Germany

**Keywords:** Total neoadjuvant therapy, TNT, Chemoradiotherapy, Rectal cancer, Complete response, Smoking

## Abstract

**Introduction:**

Complete response (CR) after total neoadjuvant therapy (TNT) in rectal cancer is linked to favorable local control and enables non-operative management (NOM). Achieving high CR rates is crucial. As no standard TNT protocol exists, we aimed to assess the impact of clinical factors and different protocols on CR rates.

**Methods:**

Rectal cancer patients undergoing TNT with curative intent between 2015 and 2024 were included in this retrospective multicenter analysis (DRKS00033000). The primary endpoint was CR. Predefined clinical and therapeutic variables were treated as covariates and evaluated as potential predictors of CR in a multivariable logistic regression model.

**Results:**

Among 245 included patients (181 men) with a median age of 62 (Q1-Q3: 54–67) years, 113 (46.1%) reached a CR. Of those, 69 (28.2%) were active smokers. Short-course radiotherapy (SCRT) was applied in 107 (43.7%) patients. Chemoradiotherapy with pyrimidine-based monotherapy or concomitant oxaliplatin was used in 65 (26.5%) and 73 (29.8%) of patients, respectively. A median of 8 (Q1-Q3: 6–9) cycles of consolidation chemotherapy was administered. The CR likelihood increased with each additional chemotherapy cycle (OR 1.19, 95%-CI: 1.04–1.38). SCRT was associated with lower CR rates (OR 0.34, 95%-CI: 0.16–0.74) compared with concomitant pyrimidine-based chemoradiotherapy. Adding concomitant oxaliplatin to 5-FU did not further increase CR rates (OR 1.06, 95%-CI: 0.50–2.27). CR was more likely in non-smokers (OR 1.92, 95%-CI: 1.03–3.57). ESMO tumor classification and treatment duration were not associated with CR.

**Conclusion:**

More intensive TNT protocols were associated with higher CR rates. Smoking cessation may be beneficial but requires external validation.

## Highlights

• Multicenter analysis evaluating predictors of complete response (CR) after TNT.
• CR improved with each chemotherapy cycle and non-smoking status.
• Adding oxaliplatin to long-course chemoradiotherapy (LCRT) did not improve CR rates.
• Short-course radiotherapy had lower CR rates than pyrimidine-based LCRT.
• Short-term distant-metastasis-free survival was improved after CR.

## Graphical abstract

**Figure:**
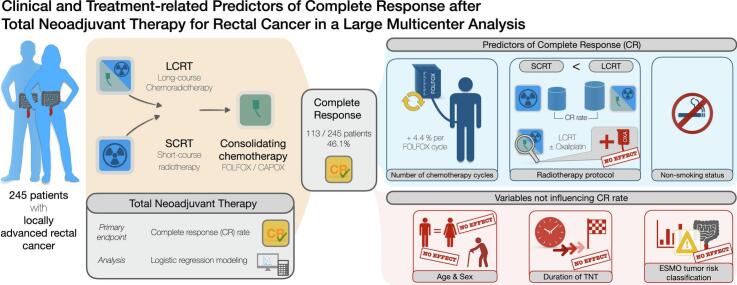

## 1 Introduction

Total mesorectal excision (TME) has long been the cornerstone of rectal cancer treatment. Recently, organ-preserving strategies have gained prominence, omitting radical surgery in selected patients who achieve a complete response (CR) after neoadjuvant therapy [1], [2], [3], [4], [5], [6]. Such non-operative management (NOM) might allow for preservation of the sphincter complex in low tumors and reduces the incidence of bowel dysfunction, such as the low anterior resection syndrome (LARS) [7]. Quality of life (QoL) was consequently better after NOM in several studies [8], [9], [10], [11], [12], [13]. Total neoadjuvant therapy (TNT), i.e., the addition of preoperative chemotherapy to neoadjuvant (chemo)radiotherapy, increases CR rates [14], [15]. TNT is, thus, increasingly used in the context of intended NOM [16]. However, no standard exists regarding the optimal TNT schedule or treatment intensity [17]. Data from current prospective trials suggest higher CR rates with an increased number of chemotherapy cycles [18]. The CAO/ARO/AIO-16 trial recently demonstrated that a prolonged interval to restaging is associated with higher CR rates, as ongoing regression was observed among patients with “near” CR [19]. Additional protocol-related factors, such as the use of concomitant chemotherapy, may be relevant, but current evidence is limited [20].

Patient-related factors, including demographic characteristics and smoking status, have likewise been associated with treatment response and even survival in rectal cancer and other malignancies [20], [21], [22], [23], [24], [25]. Therefore, we aimed to analyze the impact of these clinical and treatment-related parameters on CR rates in a large multicenter cohort encompassing various routinely applied neoadjuvant treatment protocols.

## 2 Material and methods

### 2.1 Study design and setting

We conducted a retrospective multicenter analysis within the “Young DEGRO” working group of the German Society for Radiation Oncology (DEGRO) at 23 hospitals in Germany and Austria [26], which are listed in detail in Supplement S1. The study was approved by the local ethics committee of the Faculty of Medicine at Jena University Hospital (reference number: 2023–3042-Bef, amended to allow inclusion until 2024) and by each participating center's ethics committee. The study adhered to the Declaration of Helsinki. The study protocol was prospectively registered with the German Clinical Trials Registry (DRKS, No. 00033000) and accredited by the radiation oncology working group of the German Cancer Society (Arbeitsgemeinschaft Radiologische Onkologie, ARO). All analyses were conducted in accordance with the STROBE statement [27].

Eligible patients were diagnosed with localized rectal cancer (TNM classification: T2-4 N0-2 M0/UICC stage II or III) between 2015 and 2024 and underwent neoadjuvant (chemo)radiotherapy followed by consolidation chemotherapy with curative intent. Radiotherapy was administered as hypofractionated short-course radiotherapy (SCRT), i.e., 25 Gy/5 fractions, without concomitant chemotherapy, or long-course chemoradiotherapy (LCRT) over 5–6 weeks, such as 50.4 Gy/28 fractions or 45 Gy/25 fractions with simultaneous or sequential boost and concomitant pyrimidine-based chemotherapy. Protocols included pyrimidine-based chemotherapy with intravenous 5-fluorouracil (5-FU) or oral capecitabine. As specified within the study protocol, patient and tumor characteristics with potential impact on CR rates were evaluated. Treatment characteristics comprised the applied treatment schedule, including the (chemo)radiotherapy protocol and the number of consolidation chemotherapy cycles.

### 2.2 Primary endpoint and definitions

The primary endpoint of this analysis was CR at the first evaluation after completion of TNT. It was defined as either pathological CR (pCR, in case of resection) or clinical CR (cCR). The latter required the absence of vital residual disease (TNM: ycT0 ycN0 ycM0) as determined by institutional standards at the latest three months after the end of TNT. The duration of TNT was defined as the interval between the start of treatment, i.e., the first radiotherapy fraction, and either the date of resection or, if patients entered NOM, the first restaging indicating cCR. Overall survival (OS), distant-metastasis-free survival (DMFS), and failure-free survival (FFS) were secondary endpoints, measured from the date of restaging (i.e., end of TNT) to the occurrence of the respective event or last follow-up. A detailed definition of the endpoints is provided in Supplement S2.

The tumor stage was classified according to the UICC TNM v8.0 [28]; grading was reported according to the 2019 classification of the World Health Organization (WHO) [29]. The pre-treatment tumor risk classification was categorized according to the 2017 ESMO guidelines [30]. General health condition was classified according to Karnofsky Performance Status (KPS) [31].

FOLFOX protocols are given every two weeks (q2w), whereas CAPOX is given every three weeks (q3w). We thus standardized these cycle numbers to “FOLFOX-equivalent cycles”, i.e., q2w, to allow for comparisons between different protocols (see Supplement S3 for details).

### 2.3 Statistical analysis

All analyses are exploratory. Therefore, no explicit sample size calculation was performed. Categorical variables were reported descriptively with absolute and relative frequencies (%); continuous variables with mean (±standard deviation, SD) and median (first and third quartile, Q1-Q3).

Logistic regression modelling was used to examine the association between CR and clinical factors, which were prespecified within the prospective study protocol (see Supplement S4). Variables with reported influence on tumor regression and treatment decision, including tumor stage, distance from anal verge or further risk factors (involvement of mesorectal fascia (MRF+), extramural vascular invasion (EMVI+), or positive lateral pelvic nodes), were not analyzed individually but were summarized within the ESMO risk category to reduce multicollinearity and ensure model stability. All variables were analyzed in a multivariable regression model as well as in separate univariable models for sensitivity analyses. Multicollinearity assessment was performed, and model performance was evaluated using the area under the receiver operating characteristic curve (AUC) and the Brier score [32]. Adjusted odds ratios (OR) with 95% confidence intervals (95% CI) were reported. Kaplan-Meier survival curves were generated and compared using the log-rank test; median survival was calculated using the reverse Kaplan–Meier method. For sensitivity analyses, a simplified multivariable Cox regression was performed for OS, FFS, and DMFS (Supplement S4).

A significance level of p < 0.05 was applied for all statistical tests. Logistic regression modelling of the primary endpoint was performed on complete cases from the “TNTox” cohort. For the descriptive analysis of baseline characteristics within this subset, missing values were handled using pairwise deletion, resulting in varying numbers of observations across variables. All analyses were conducted with SPSS 29.0 (IBM SPSS Statistics, Armonk, NY/USA) as well as JASP v0.95.4 (JASP Team, 2025, [33]).

## 3 Results

A total of 245 patients with a median age at diagnosis of 62 (Q1-Q3: 54–67) years were included. Among them, 181 (73.9%) were male. Most patients had good performance status (median KPS of 90%). An active smoking status was reported for 69 patients (28.2%). Most of the patients presented with tumors classified as “advanced” (141 patients/57.6%) or “bad” (48/19.6%) according to the 2017 ESMO risk category. LCRT was administered either with pyrimidine-based concomitant monotherapy in 65 patients (26.5%) or with concomitant 5-FU and oxaliplatin in 73 patients (29.8%). SCRT was used in the remaining 107 patients (43.7%). A median of 8 (Q1-Q3: 6–9) cycles of consolidation chemotherapy were administered. FOLFOX (163 patients/66.5%) and CAPOX (78 patients/31.8%) were the most commonly applied schedules. The patient and treatment characteristics are summarized in Table 1.

**Table 1:** Patient and treatment characteristics. (A) Categorical variables are presented as absolute (n) and relative (%) frequencies for patient and treatment characteristics. (B) Continuous variables are presented as mean ± standard deviation (SD) and median with first (Q1) and third (Q3) quartiles. Due to rounding, percentages may not total 100%. Missing data were treated using pairwise deletion, resulting in different numbers of included cases per variable. Abbreviations: circumferential resection margin (CRM), extramural vascular invasion (EMVI), European Society for Medical Oncology (ESMO), Long-course chemoradiotherapy (LCRT), Short-course radiotherapy (SCRT).

(A)		
Baseline characteristics		
Sex		
(N = 245)	n	%
Female (f)	64	26.1
Male (m)	181	73.9
Tumor localization		
(N = 245)	n	%
0–6 cm from anal verge	133	54.3
6–12 cm from anal verge	106	43.3
>12 cm from anal verge	6	2.4
cT stage		
(N = 245)	n	%
T1	3	1.2
T2	14	5.7
T3 a/b	105	42.9
T3 c/d	64	26.1
T4	59	24.1
cN stage		
(N = 245)	n	%
N0	21	8.6
N1	79	32.2
N2	101	41.2
N+	44	18.0
Grading		
(N = 232)	n	%
G1	12	5.2
G2	211	90.9
G3	9	3.9
Involvement of lateral pelvic nodes		
(N = 234)	n	%
No	175	74.8
Yes	59	25.2
Extramural vascular invasion		
(N = 207)	n	%
EMVI-	144	69.6
EMVI+	63	30.4
Involved mesorectal fascia		
(N = 238)	n	%
MRF involved (< 1 mm)	116	48.7
MRF threatened (1–2 mm)	26	10.9
MRF clear (> 2 mm)	96	40.3
ESMO risk category		
(N = 245)	n	%
early (good)	8	3.3
intermediate	48	19.6
bad	48	19.6
advanced (ugly)	141	57.6
Active smoker		
(N = 245)	n	%
No	176	71.8
Yes	69	28.2
Treatment characteristics		
Chemoradiotherapy		
(N = 245)	n	%
SCRT	107	43.7
LCRT	138	56.3
of those		
LCRT, 5-FU monotherapy	61	24.9
LCRT, 5-FU/Oxaliplatin	77	31.4
Radiotherapy fractionation schedule		
(N = 245)	n	%
Hypofractionated, i.e., 25.0 (5.0) Gy	107	43.7
Normofractionated, i.e., 50.4 (1.8) Gy or 50.0 (2.0) Gy	111	45.3
Normofractionated + boost concept (i.e., 45.0 Gy + Boost)	20	8.2
Normofractionated + boost (i.e., 50.4 Gy + Boost)	6	2.4
Other schedule ^1)^	1	0.4
Consolidation chemotherapy protocol		
(N = 245)	n	%
FOLFOX	163	66.5
CAPOX	78	31.8
Other protocol ^2)^	4	1.6
Complete response		
(N = 245)	n	%
no CR	132	53.9
clinical CR	59	24.1
pathological CR	54	22.0

(B)						
	n	Mean	Std. Deviation	Median	Q1	Q3
Age (years)	245	60.4	10.3	62	54	67
KPS (%)	243	90.7	8.9	90	90	100
Number of consolidation chemotherapy cycles ^3)^	245	7.1	2.3	8	6	9
Duration of TNT (months)	245	6.5	2.3	6.3	5.4	7.3

Footnotes:

1) Other fractionation schedules:

45.9 Gy (27 fractions) + simultaneous integrated boost to GTV: n = 1.

2) other protocols of sequential chemotherapy:

5-FU monotherapy: n = 2.

FOLFOXIRI: n = 1.

FOLFIRINOX: n = 1.

3) standardized to FOLFOX-equivalents (q2w).

**Fig. 1:**
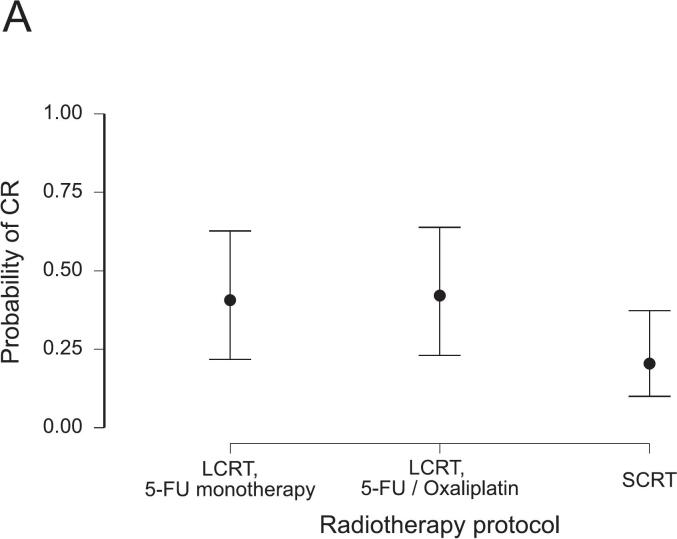

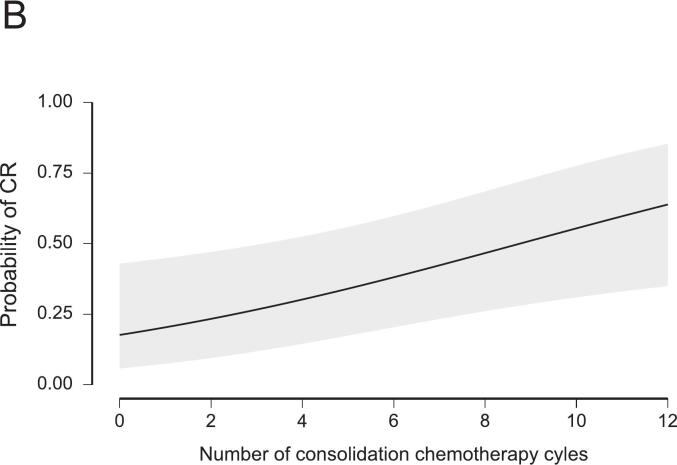

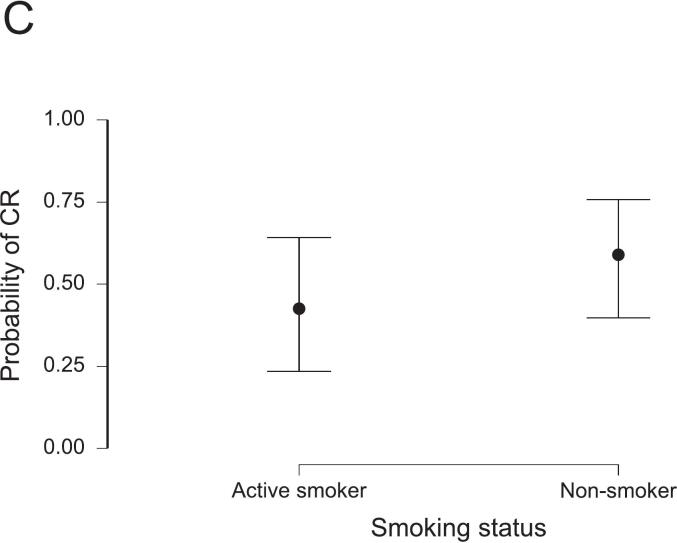
Predicted probability of complete response (CR) as a function of radiotherapy protocol (A), number of consolidation chemotherapy cycles (B), and smoking status (C), derived from the logistic regression model. See also Table 2 and Supplement S3 for model specifications.

A CR was reached in 113 patients (46.1%). Among them, 59 patients (24.1%) had a clinical CR and were managed non-operatively. The influence of the prespecified variables on CR rates was evaluated using logistic regression models, summarized in Table 2. Demographic factors, including age and sex, did not significantly influence CR rates in the uni- and multivariable regression models. Non-smoking status was associated with increased CR rates (OR 1.92, 95%-CI: 1.03–3.57). Tumor characteristics, as summarized per ESMO risk category, did not influence CR rates in the multivariable model. However, “intermediate” risk tumors had a significantly increased likelihood of CR compared with “advanced” tumors (OR 2.06, 95%-CI: 1.06–4.02) in the univariable model. Regarding the treatment protocols, we observed a strong effect of concomitant LCRT. A CR was less likely after SCRT compared with LCRT using pyrimidine-based monotherapy (OR 0.38, 95%-CI: 0.17–0.82). Adding oxaliplatin to LCRT was not associated with higher CR rates (OR 1.06, 95%-CI: 0.50–2.27). The duration of TNT showed no significant association with CR. However, the number of consolidation chemotherapy cycles was associated with higher odds for CR (OR 1.19, 95%-CI: 1.04–1.38). Notably, this effect reached significance only in the multivariable, but not in the univariable model (OR 1.06, 95%-CI: 0.95–1.18). Given a baseline CR rate of 46.1%, the addition of one FOLFOX cycle increased the estimated probability of a CR to 50.5%, corresponding to an absolute risk increase of 4.4% (Equation (1). The predicted probabilities of CR depending on the significant influencers in this model are presented in Fig. 1. The model performance was moderate, with an AUC of 0.69 and a Brier score of 0.22. Multicollinearity was negligible (all variance influence factors, VIF < 1.66), and overall model fit was modest (Nagelkerke R2 0.14). A detailed summary of the model’s performance metrics is provided in Supplement S5.

**Table 2:** Results from logistic regression analyses for the achievement of complete response in relation to prespecified clinical variables. Adjusted odds ratios (ORs) with 95% confidence intervals (CIs) and p-values are presented from uni- and multivariable regression models. The number of patients (n) included after casewise deletion is provided.

	Univariable logistic regression model (n = 245)				Multivariable logistic regression model (n = 245)			
Variable	Odds Ratio	Lower 95% CI	Upper 95% CI	p	Odds Ratio	Lower 95% CI	Upper 95% CI	p
Age (in years)	1.01	0.99	1.04	0.402	1.00	0.97	1.03	0.963
Sex (male)	1.14	0.64	2.02	0.658	1.04	0.56	1.95	0.892
ESMO risk category (bad)	0.81	0.41	1.59	0.539	0.82	0.40	1.66	0.578
ESMO risk category (intermediate)	2.06	1.06	4.02	0.034	1.76	0.87	3.58	0.119
ESMO risk category (early)	4.05	0.79	20.77	0.094	4.38	0.77	24.87	0.096
TNT duration (in months)	1.12	0.99	1.26	0.067	1.02	0.90	1.17	0.735
Radiotherapy protocol (LCRT, 5-FU/Oxaliplatin)	0.85	0.43	1.66	0.628	1.06	0.50	2.27	0.878
Radiotherapy protocol (SCRT)	0.39	0.21	0.75	0.005	0.38	0.17	0.82	0.014
Number of consolidation chemotherapy cycles	1.06	0.95	1.18	0.330	1.19	1.04	1.38	0.015
Non-smoker	1.92	1.08	3.42	0.027	1.92	1.03	3.57	0.041

**Fig. 2:**
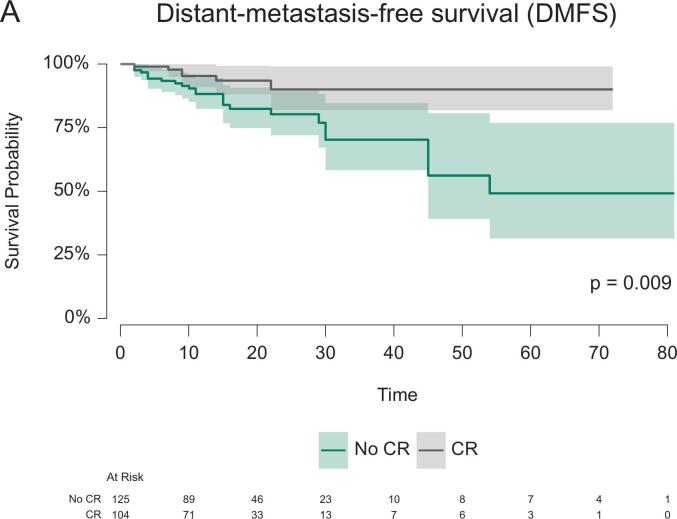

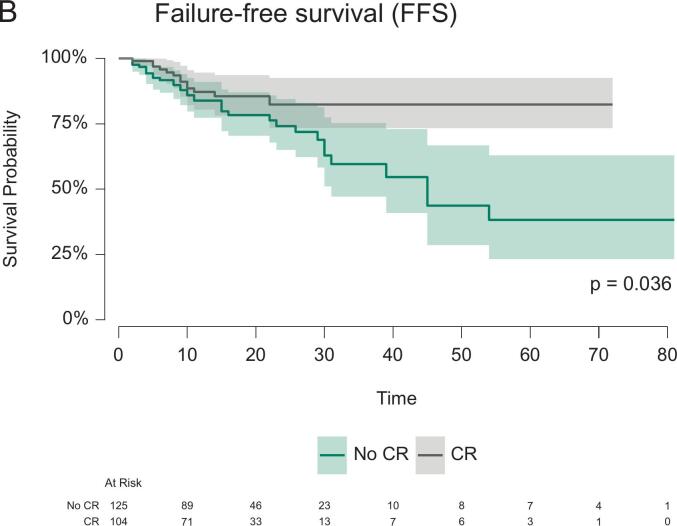

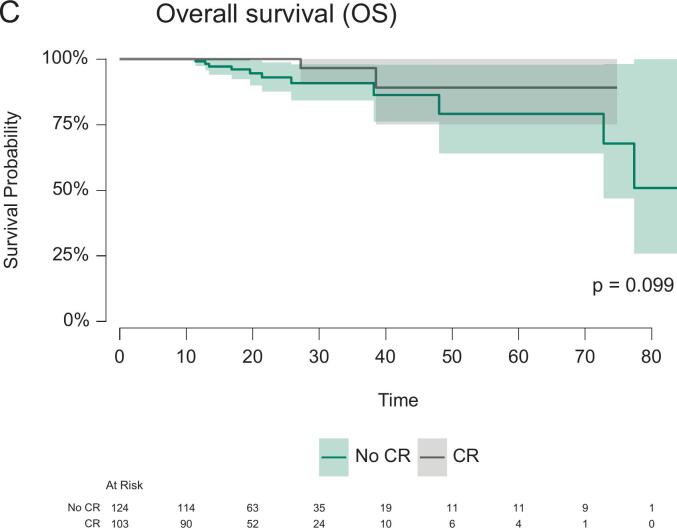
(A) Distant-metastasis-free survival, (B) Failure-free survival (FFS) and (C) overall survival (OS) curves stratified by complete response (CR) status. The survival probabilities are accompanied by the respective 95% confidence intervals (CI). The number of patients at risk is provided for both treatment groups below the plot. The p-value (p) from the log-rank test is provided. Due to missing survival information, 16 patients were excluded from DMFS and FFS analyses as well as 18 patients from the OS analysis.

A relapse was reported for 46/186 patients (19.8%, see also Table 3) within short-term follow-up (median follow-up 17 (95%-CI: 15–20) months). Among them, 30 patients experienced distant metastasis. Local recurrence was documented in 16 patients, which occurred in 9 of 178 patients after resection and in 7 of 53 patients during NOM after cCR. No local recurrence after pCR and resection was reported. Death occurred in 13 of 216 patients (5.7%). The OS, FFS, and DMFS curves, stratified by CR status, are shown in Fig. 2. At 18 months follow-up, the OS, FFS and DMFS for patients with versus without CR were 100.0% (95%-CI: 100.0%-100.0%) versus 96.1% (95%-CI: 92.3%-99.9%), 85.6% (95%-CI: 78.2%-93.6%) versus 78.3% (95%-CI: 70.4%-87.1%) and 93.5% (95%-CI: 88.1%-99.3%) versus 82.4% (95%-CI: 74.9%-90.7%), respectively. The log rank test indicated significant differences in the FFS (p = 0.036) and DMFS (p = 0.009) curves stratified by CR. CR remained a significant predictor only of DMFS (Hazard ratio, HR 0.39, 95%-CI 0.16–0.97, p = 0.043) in the multivariable Cox regression. There was no significant association between CR and OS (p = 0.139) or FFS (p = 0.095). ESMO risk category “advanced” tumors were associated with worse FFS (HR 2.23, 95%-CI 1.17–4.32, p = 0.014) and DMFS (HR 3.03, 95%-CI 1.30–7.10, p = 0.011). Detailed results of these secondary regression models are provided in Table 4 as well as Supplement S6.

**Table 3:** Descriptive statistics with absolute (n) and relative frequencies (%) of recurrence patterns, stratified by complete response (CR) and organ preservation. The number of patients with available follow-up data per stratum (N) is given. Note that multiple responses for the localization of recurrence per patient were possible.

		All patients (CR and no CR)		Patients with CR (cCR and pCR combined)		Patients entering organ preservation (after cCR)	
N		231		106		53	
		n	%	n	%	n	%
Patients with recurrence		46	19.9%	13	12.3%	9	17.0%
*of those*							
	Local recurrence, in-field	16	6.9%	7	6.6%	7	13.2%
	Local recurrence, out-of-field	3	1.3%	2	1.9%	1	1.9%
	Distant recurrence (metastasis)	30	13.0%	6	5.7%	3	5.7%
	No relapse	185	80.1%	93	87.7%	44	83.0%

**Table 4:** Estimated hazard ratios (HRs) from multivariable Cox regression analyses for overall survival (OS), failure-free survival (FFS), and distant-metastasis-free survival (DMFS), including 95% confidence intervals (CIs) and p-values.

Overall Survival (OS)					
	N = 227	Hazard Ratio	Lower 95% CI	Upper 95% CI	p
	Age (years)	1.00	0.94	1.06	0.880
	Male sex	1.54	0.40	5.84	0.530
	ESMO risk category “advanced”	2.09	0.61	7.17	0.239
	Complete response	0.31	0.07	1.46	0.139

Failure-free survival (FFS)					
	N = 229	Hazard Ratio	Lower 95% CI	Upper 95% CI	p
	Age (years)	0.98	0.95	1.00	0.068
	Male sex	1.09	0.56	2.12	0.796
	ESMO risk category “advanced”	2.23	1.17	4.23	0.014
	Complete response	0.58	0.30	1.10	0.095

Distant-metastasis-free survival (DMFS)					
	N = 229	Hazard Ratio	Lower 95% CI	Upper 95% CI	p
	Age (years)	0.96	0.93	0.99	0.015
	Male sex	0.95	0.43	2.09	0.891
	ESMO risk category “advanced”	3.03	1.30	7.09	0.011
	Complete response	0.39	0.16	0.97	0.043

## 4 Discussion

To the best of our knowledge, this retrospective multicenter cohort represents one of the largest datasets evaluating patient characteristics and varying treatment protocols. A current systematic review elaborated different biochemical predictors, patient demographics, and treatment protocols with influence on CR rates from 9 studies, but the overall quality of evidence was low to very low [20]. To date, the optimal chemotherapy sequence within TNT has been evaluated within the prospective OPRA and CAO/ARO/AIO-12 trials, demonstrating that consolidation chemotherapy is superior regarding CR rates [34], [35]. Thus, only consolidation chemotherapy was allowed per inclusion criteria in our cohort in order to reduce inhomogeneity and ease the evaluation of additional factors. The optimum duration and number of chemotherapy cycles are still unclear. Higher CR rates were seen within the OPRA trial with longer consolidation chemotherapy compared to the CAO/ARO/AIO-12 trial. However, survival was similar in a pooled comparison [36]. As demonstrated by the TIMING trial, a higher number of chemotherapy cycles also improved CR rates [18]. An increasing number of chemotherapy cycles coincidentally extends the overall duration of TNT, complicating causal attribution. Gani et al. demonstrated an increased overall CR rate after longer follow-up, as ongoing tumor regression was often seen in patients with “near CR” at first follow-up [19]. These findings cannot easily be extrapolated to a general patient population, as a positive selection of “treatment responders” was performed by longitudinal response evaluation. Consistent with the TIMING trial, CR rates were associated with the number of chemotherapy cycles in our cohort. Nevertheless, the observed effects were small and need to be weighed against additional toxicity in case of intended NOM. Especially, oxaliplatin-related peripheral neuropathy is a common dose-dependent toxicity and might negatively impact QoL [37], [38].

As demonstrated earlier, CR was more likely after LCRT in this cohort [39]. However, LCRT was not associated with increased toxicity or differences in survival in our prior analysis. We observed that the addition of oxaliplatin to LCRT did not increase the likelihood of CR. For neoadjuvant CRT, additional oxaliplatin was associated with a disease-free survival (DFS) benefit and increased pCR rates in several trials; OS was not improved [40], [41], [42]. For example, concomitant oxaliplatin was associated with improved DFS and OS in patients <60 years in a secondary analysis of the CAO/ARO/AIO-04 trial [43]. This protocol was also implemented as part of the TNT protocols at several centers within the current cohort. On the downside, an increasing toxicity rate might be observed during LCRT [40], [41], [42]. A recent randomized trial assessed three cycles of CAPOX or capecitabine with LCRT. Comparable to our findings, CAPOX did not increase CR rates, but tumor control was superior in patients with (near)-CR [44]. For TNT protocols, no uniform standard has been established to date. Larger cohorts and longer follow-up are required to determine whether concomitant oxaliplatin provides any incremental benefit during LCRT.

Active smoking has been identified as a negative prognostic factor across multiple cancer types, including rectal cancer in particular [21], [22], [45]. Moreover, a *meta*-analysis reported that smoking cessation may yield outcomes comparable to those of never smokers [22]. Herein, non-smoking status during treatment was associated with higher CR rates. This observation may be relevant for patient counselling, although external validation is still required. Former smoking status was not recorded, but may be relevant for further analyses.

We did not observe any significant effects of age or sex, which is consistent with the findings of Yilmaz et al. [46]. Similarly, Zorcolo et al. reported no impact of either variable on CR rates during neoadjuvant CRT [47]. In contrast, two retrospective cohorts found that early-onset rectal cancer was associated with higher CR rates following TNT [48], [49].

Secondary survival analyses demonstrated improved DMFS in patients achieving a CR; however, follow-up was relatively short. CR is unlikely to be the sole determinant of outcome. For example, given that minimal residual disease is thought to drive distant relapse [50], [51], [52], CR may still function as a surrogate marker of treatment efficacy at the individual level, potentially indicating effective clearance of MRD. In a larger cohort, McDermott et al. reported an OS benefit for CR patients compared with non-CR patients following TNT [53]. This effect was not observed in our cohort, but the number of observed events was very small. After neoadjuvant CRT, a *meta*-analysis did not demonstrate an OS advantage for CR [54].

Note that the presented cohort included patients who underwent radical resection as well as patients who entered a watch-and-wait program. Local regrowth is a relatively common observation during watch and wait approaches [4]. Resection with pCR is associated with very good local control [55], [56]. Therefore, local recurrence rates were not directly compared between patients with and without CR. The small number of relapses within the limited follow-up period did not allow for further stratification by type of CR (cCR versus pCR).

Beyond these clinical parameters, individual morphological and biological factors, such as MRI radiomics features and liquid-biopsy–derived markers including circulating tumor DNA, have also been reported to predict CR [57], [58], [59], [60], [61], [62]. Such features and the relatively poor performance of several prediction models based on clinical parameters might indicate that individual tumor biology could be more relevant for the prediction of tumor response. Biology- and response-driven treatment tailoring might thus be a promising approach to avoid over- and undertreatment, especially in the light of intended NOM [63]. Nevertheless, these features must still be considered experimental and have not yet been introduced in routine clinical practice.

### 4.1 Strengths and limitations

A total of 245 patients treated at 23 high-volume institutions in Germany and Austria were included in this multicenter analysis, offering a representative real-world population managed with current TNT regimens. All inclusion parameters and statistical procedures were outlined beforehand in the study protocol, ensuring a transparent methodological setup. The consistent therapeutic sequence, i.e., LCRT or SCRT followed by consolidation chemotherapy, helped to curtail variability between participating centers. In addition, the regression models accounted for a priori defined confounders, reducing the risk of bias and reinforcing the credibility of the reported associations.

Nevertheless, several limitations must be acknowledged. As a retrospective, non-randomized analysis, causal inference is limited and the results should be interpreted as hypothesis-generating. The selection of SCRT versus LCRT was not standardized due to the study design. Consequently, confounding by indication is likely, particularly with respect to a possible preference of LCRT for NOM, or SCRT for timely subsequent systemic therapy in high-risk patients. No external validation of the regression model was realized, and no internal split into training and validation cohorts was performed because of the limited sample size. Given the exploratory nature of the study, we prioritized using the whole cohort for model development to maximize statistical power. Therefore, the regression model was primarily intended for exploratory analyses and adjustment for relevant confounders, rather than for clinical prediction. The model performance was modest, with a Nagelkerke R2 of 0.14, indicating that it explained only a limited proportion of outcome variability. This is likely attributable to heterogeneity in the cohort, particularly within the ESMO risk categories, which may encompass tumors with diverse biological behavior and response patterns. Including the ESMO risk category’s features separately within the model resulted in relevant model instability related to multicollinearity issues. Given the relatively short follow-up and the limited number of events, multivariable Cox modelling did not permit inclusion of an extensive set of covariates. With these hypothesis-generating survival results, larger cohorts with longer follow-up would be needed to address these secondary questions and to allow relevant subgroups (cCR versus pCR) being stratified.

## 5 Conclusion

CR rates were associated with the intensity of the applied TNT protocol, comprising the use of concomitant LCRT and a higher number of chemotherapy cycles, in this cohort. Adding oxaliplatin to concomitant CRT did not significantly increase the CR rates compared to pyrimidine-based monotherapy. Non-smoking status was associated with higher CR rates. Smoking cessation might thus be recommended to patients, but external validation is warranted. CR was associated with improved short-term DMFS in this cohort.

Eq. (1) given the baseline probability of a complete response (P_0_ = 0.461) and the Odds Ratio (OR) of 1.194 for an additional FOLFOX cycle of consolidation chemotherapy, ΔP was calculated using the following formulas 1–4.

(1) $${\mathrm{Odds}}_{0}=\frac{P_{0}}{1-P_{0}}andOR=\frac{{\mathrm{Odds}}_{1}}{{\mathrm{Odds}}_{0}}$$

(2) $$P_{1}=\frac{{\mathrm{Odds}}_{1}}{1+{\mathrm{Odds}}_{1}}=\frac{{\mathrm{OR}\times Odds}_{0}}{1+{\mathrm{OR}\times Odds}_{0}}=\frac{\mathrm{OR}\times \frac{P_{0}}{1-P_{0}}}{1+OR\times \frac{P_{0}}{1-P_{0}}}=0.505$$

(3) $$\Delta P=P_{1}-P_{0}=0.505-0.461$$

(4) $$\Delta P=\frac{\mathrm{OR}\times \frac{P_{0}}{1-P_{0}}}{1+OR\times \frac{P_{0}}{1-P_{0}}}-P_{0}=0.044$$

## Declaration of Competing Interest

The authors declare the following financial interests/personal relationships which may be considered as potential competing interests: Felix Ehret reports a relationship with ZAP Surgical Systems, Inc. that includes: consulting or advisory and travel reimbursement. Felix Ehret reports a relationship with Accuray Inc that includes: consulting or advisory, funding grants, and travel reimbursement. Felix Ehret reports a relationship with German Cancer Aid that includes: funding grants. Alexander Ruehle reports a relationship with Novocure Inc that includes: funding grants, speaking and lecture fees, and travel reimbursement. Alexander Ruehle reports a relationship with Johnson & Johnson and Need Inc that includes: consulting or advisory. Alexander Ruehle reports a relationship with Merck Healthcare Germany GmbH that includes: speaking and lecture fees. Alexander Ruehle reports a relationship with AstraZeneca that includes: speaking and lecture fees. Alexander Fabian reports a relationship with Merck Sharp & Dohme Corp that includes: consulting or advisory. If there are other authors, they declare that they have no known competing financial interests or personal relationships that could have appeared to influence the work reported in this paper.
