# Supplementary material for: Clinical and treatment-related predictors of complete response after total neoadjuvant therapy for rectal cancer in a large multicenter analysis
Source: Clin Transl Radiat Oncol. 2026 Feb 5;58:101120. doi: 10.1016/j.ctro.2026.101120 (PMC12907501; doi:10.1016/j.ctro.2026.101120)
Supplement: Supplementary Data 1 [file mmc1.pdf]

Supplementary Document: Clinical and Treatment-related Predictors of Complete Response after Total Neoadjuvant Therapy for Rectal Cancer in a Large Multicenter Analysis

Georg W. Wurschi, Melanie Schneider, Jan-Niklas Becker, Bernd Frerker, Samuel Vorbach, Felix Ehret, Markus Diefenhardt, Fabian Schunn, Maria-Elena von Gruben, Marcel Büttner, Elgin Hoffmann, Alexander Rühle, Josephine Beier, Simone Ferdinandus, Maike Trommer, Ezgi Ceren Sahin, Julian Hlouschek, Kynann Aninditha, Daphne Schepers von Ohlen, Justus Kaufmann, Alina Depardon, Hai Minh Ha, Simon Trommer, Christopher Kessler, Adrianna Cieslak, Alexander Fabian, Florian Rißner, Mathias Sonnhoff, Maximilian Römer and Klaus Pietschmann

Table of contents

S1. Table: Participating centers 2

S2. Definition of endpoints 3

S3. Equation: Standardization of chemotherapy cycles 4

S4. Table: Specification of regression models 5

S5. Performance metrics for the multivariable regression model 6

S6. Table: Test of proportional hazards assumption for Cox regression models 7

References 8

S1. Table: Participating centers

Center	Number of Patients
Jena University Hospital (Jena / Germany)	36
University Hospital Carl Gustav Carus Dresden (Dresden / Germany)	26
Medical University of Innsbruck (Innsbruck / Austria)	23
Rostock University Medical Center (Rostock / Germany)	22
Charité – Universitätsmedizin Berlin (Berlin / Germany)	21
University Hospital Goethe University Frankfurt (Frankfurt (Main) / Germany)	18
Hannover Medical School (Hannover / Germany)	16
University Hospital Heidelberg (Heidelberg / Germany)	11
Faculty of Medicine and University Hospital Cologne (Köln / Germany)	11
University Hospital Tübingen (Tübingen / Germany)	9
West German Cancer Center, University Hospital Essen (Essen / Germany)	8
University Hospital Hamburg-Eppendorf (Hamburg / Germany)	7
University Medical Center Schleswig-Holstein / Campus Lübeck (Lübeck / Germany)	7
University Medical Center Leipzig (Leipzig / Germany)	6
University Medical Center of the Johannes-Gutenberg-University Mainz (Mainz / Germany)	6
Otto von Guericke Universität Magdeburg (Magdeburg / Germany)	5
Klinikum Stuttgart (Stuttgart / Germany)	4
University Hospital Halle (Halle (Saale) / Germany)	3
Universitätsklinikum Erlangen, Friedrich-Alexander-Universität Erlangen-Nürnberg (Erlangen / Germany)	2
Technical University of Munich (TUM), School of Medicine and Klinikum Rechts der Isar (München / Germany)	2
University Medical Center Schleswig-Holstein / Campus Kiel (Kiel / Germany)	1
University Medicine Mannheim, Medical Faculty Mannheim (Mannheim / Germany)	1
Strahlentherapie Bremen (radiation oncology practice, Bremen / Germany)	1
Total	245

S2. Definition of endpoints

Primary endpoint

The primary endpoint was complete response (CR). According to the study protocol, CR was defined as a composite endpoint comprising pathological CR and clinical CR

A pathological CR was defined as resection with histological evidence of complete tumor regression, corresponding to Dworak tumor regression grade 4 [1].

A clinical CR was defined as the absence of any evidence of viable residual disease at the latest three months after completion of total neoadjuvant therapy (TNT). Restaging to confirm a clinical complete response was performed according to institutional standards and, per protocol, required at minimum a rectoscopy and a pelvic magnetic resonance imaging (MRI).

In cases where non-operative management was initiated at the physician's discretion due to a near CR (i.e., a small ulcer or hyperplastic scar) at the first follow-up, and a subsequent follow-up within three months after the end of TNT demonstrated further tumor regression without evidence of residual disease, patients were classified as having achieved clinical CR. For these patients, the time point of the follow-up examination confirming CR was used for subsequent analyses.

Survival endpoints

Overall survival (OS), distant-metastasis-free survival (DMFS), and failure-free survival (FFS) were analyzed. All survival endpoints were measured from the date of restaging (i.e., completion of TNT) to the occurrence of the respective event or last follow-up and were estimated using the Kaplan–Meier method. No events occurred during therapy, making this approach appropriate to avoid immortal-time bias.

- Overall survival (OS) was defined as the time from restaging to death from any cause. Patients alive at last follow-up were censored.
- Distant-metastasis-free survival (DMFS) was defined as the time from restaging to the occurrence of distant metastatic disease, including metastases to non-regional lymph nodes (M1 according to UICC TNM version 8.0 [2]), or death from any cause, whichever occurred first. Patients without distant metastases at last follow-up were censored.
- Failure-free survival (FFS) was defined as the time from restaging to the first occurrence of disease-related failure, including local regrowth or recurrence, distant metastasis, or death from any cause, whichever occurred first. Patients without recurrence at last follow-up were censored.

S3. Equation: Standardization of chemotherapy cycles

As standard FOLFOX cycles are given every two weeks (q2w) and CAPOX (q3w) every three weeks, respectively, a standardization of cycle numbers was required to allow for comparison regarding the number of consolidation chemotherapy cycles. Standardization of n_{CapOx} to n_{FOLFOX} was performed as follows:

$$n_{FOLFOX-equivalent} = n_{CAPOX} * \frac{3}{2}$$

S4. Table: Specification of regression models

For the primary endpoint, a multivariable regression model was constructed (A). Short-term survival data (secondary endpoint) were evaluated with Kaplan-Meier curves stratified by CR rate. Additional exploratory Cox regression models were built for sensitivity analyses (B). Abbreviations: 5-fluorouracil, 5-FU; distant-metastasis free survival, DMFS; failure-free survival, FFS; long-course chemoradiotherapy, LCRT; overall survival, OS.

**A** Primary endpoint	Potential risk factors
Complete response (CR)	• Age (per additional year)<br>• Sex (male versus female)<br>• Number of consolidation chemotherapy cycles (per cycle) *<br>• Duration of TNT (per additional month) **<br>• Radiotherapy protocol (reference: LCRT with 5-FU monotherapy)<br>• Current smoking status (non-smoker versus active smoker)<br>• ESMO risk category (reference: ‘advanced’ tumors)
**B** Secondary endpoints	Potential risk factors
OS / FFS / DMFS	• Age (per additional year)<br>• Sex (male versus female)<br>• ESMO risk category (‘advanced’ tumors versus lower-risk tumors, binary)<br>• Complete response (CR versus no CR)

* Standardized to FOLFOX-cycles (q2w)
** Interval between start of treatment and last restaging (watch and wait) or resection.

S5. Performance metrics for the multivariable regression model

Additional performance metrics and model diagnostics for the multivariable logistic regression model predicting complete response (CR). Included are classification metrics (area under the receiver-operator characteristic curve, AUC, and Brier score), pseudo-R^2 values (Nagelkerke R^2), and multicollinearity diagnostics (tolerance, variance inflation factor).

**Performance metrics**	
	Value
Nagelkerke R^2	0.144
AUC	0.692
Brier score	0.221

**Multicollinearity Diagnostics**		
	Tolerance	VIF
Age	0.906	1.103
Duration of TNT	0.783	1.277
Number of consolidation chemotherapy cycles	0.692	1.446
Sex	0.946	1.058
Smoking status	0.930	1.076
ESMO tumor risk category	0.860	1.162
Radiotherapy protocol	0.601	1.664

S6. Table: Test of proportional hazards assumption for Cox regression models

Proportional hazards assumption test tables of the Cox regression models (Table 3) for overall survival (OS, A), failure-free survival (FFS), and distant-metastasis-free survival (DMFS, C). The proportional hazards assumption was not violated (all tests p > 0.05).

**(A) OS**			
	Chi Square	df	p
Complete response (CR)	1.90	1	0.168
Male sex	1.31	1	0.252
ESMO risk category (Advanced risk)	2.86	1	0.091
Age (years)	3.00	1	0.083
Global	5.81	4	0.213

**(B) FFS**			
	Chi Square	df	p
Complete response (CR)	0.05	1	0.824
Male sex	0.14	1	0.710
ESMO risk category (Advanced risk)	2.09	1	0.149
Age (years)	0.07	1	0.797
Global	2.79	4	0.594

**(C) DMFS**			
	Chi Square	df	p
Complete response (CR)	0.62	1	0.431
Male sex	0.15	1	0.698
ESMO risk category (Advanced risk)	1.88	1	0.171
Age (years)	0.10	1	0.757
Global	3.46	4	0.484

References

1. Dworak, O., L. Keilholz, and A. Hoffmann, *Pathological features of rectal cancer after preoperative radiochemotherapy.* Int J Colorectal Dis, 1997. **12**(1): p. 19-23.
2. Brierley, J.D., M.K. Gospodarowicz, and C. Wittekind, *TNM classification of malignant tumours*. 2017: John Wiley & Sons.
